# Single Balloon Enteroscopy-Assisted ERCP Using Rendezvous Technique for Sharp Angulation of Roux-en-Y Limb in a Patient with Bile Duct Stones

**DOI:** 10.1155/2009/154084

**Published:** 2010-02-14

**Authors:** Takao Itoi, Kentaro Ishii, Atsushi Sofuni, Fumihide Itokawa, Toshio Kurihara, Takayoshi Tsuchiya, Shujiro Tsuji, Junko Umeda, Fuminori Moriyasu

**Affiliations:** Department of Gastroenterology and Hepatology, Tokyo Medical University, Tokyo 160-0023, Japan

## Abstract

The acute angulation of Roux-en-Y (R-Y) limb precludes endoscopic access for endoscopic retrograde cholangiopancreatography (ERCP) even using a balloon enteroscopy. Here, we describe a case of successful single balloon enteroscopy (SBE)-assisted ERCP using a rendezvous technique in a patient with sharply angulated R-Y limb in a 79-year-old woman who had bile duct stones. *Method.* At first, a guidewire was passed antegradely through the major papilla after the needle puncture using percutaneous transhepatic biliary drainage technique. A hydrophilic guidewire with an ERCP catheter was antegradely advanced beyond the Roux limb. After a guidewire was firmly grasped by a snare forceps, it was pulled out of the body, resulting that the enteroscope could advance to the papilla. After papillary dilation, complete removal of bile duct stones was achieved without any procedure-related complication. In conclusion, although further study is needed, SBE-assisted ERCP using a rendezvous technique may have a potential for selected patients.

## 1. Introduction

The acute angulation of Roux-en-Y (R-Y) limb preclude endoscopic access for endoscopic retrograde cholangiopancreatography (ERCP) [1, 2] using even the balloon enteroscopy [3–10]. Here, we describe a case of successful single balloon enteroscopy (SBE)-assisted lithotripsy using a rendezvous technique in a patient with sharply angulated R-Y limb.

## 2. Case Report

**Figure 1:**
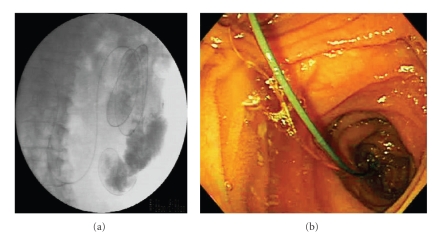
Rendezvous technique for acute angulation of Roux-en-Y limb. (a) X-ray film showed that a guidewire advanced over the Roux-en-Y limb. (b) Endoscopic imaging revealed that a guidewire reached the Roux-en-Y limb.

A 79-year-old woman who had total gastrectomy, with R-Y for gastric cancer, was admitted for the treatment of bile duct stones. Although we tried SBE-assisted ERCP (XSIF-Q260Y; Olympus Medical Systems, Tokyo, Japan), an enteroscope could not be advanced to sharply angulated R-Y limb. Three days later, we performed rendezvous technique-assisted SBE using carbon dioxide during the procedure. At first, a guidewire was passed antegradely through the major papilla after the needle puncture using a percutaneous transhepatic biliary drainage (PTBD) technique. A hydrophilic guidewire (Radifocus, Terumo, Tokyo, Japan) with an ERCP catheter was antegradely advanced beyond the Roux limb (Figures 1(a) and 1(b)). 

**Figure 2:**
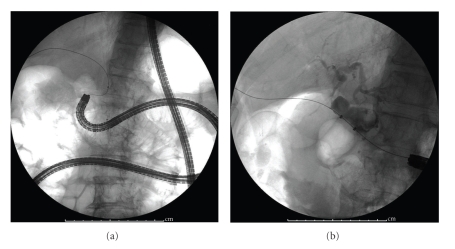
Enteroscope assisted ERCP. (a) X-ray film showed that an enteroscope reached the papilla using rendezvous technique. (b) Cholangiogram revealed bile duct stones.

Then the enteroscope was inserted to the Roux limb after a guidewire was found and firmly grasped by a snare forceps, it was pulled out of the body through the working channel of the enteroscope resulting that the enteroscope could advance to the papilla (Figure 2(a)). Cholangiogram revealed bile duct stones (Figure 2(b)). 

**Figure 3:**
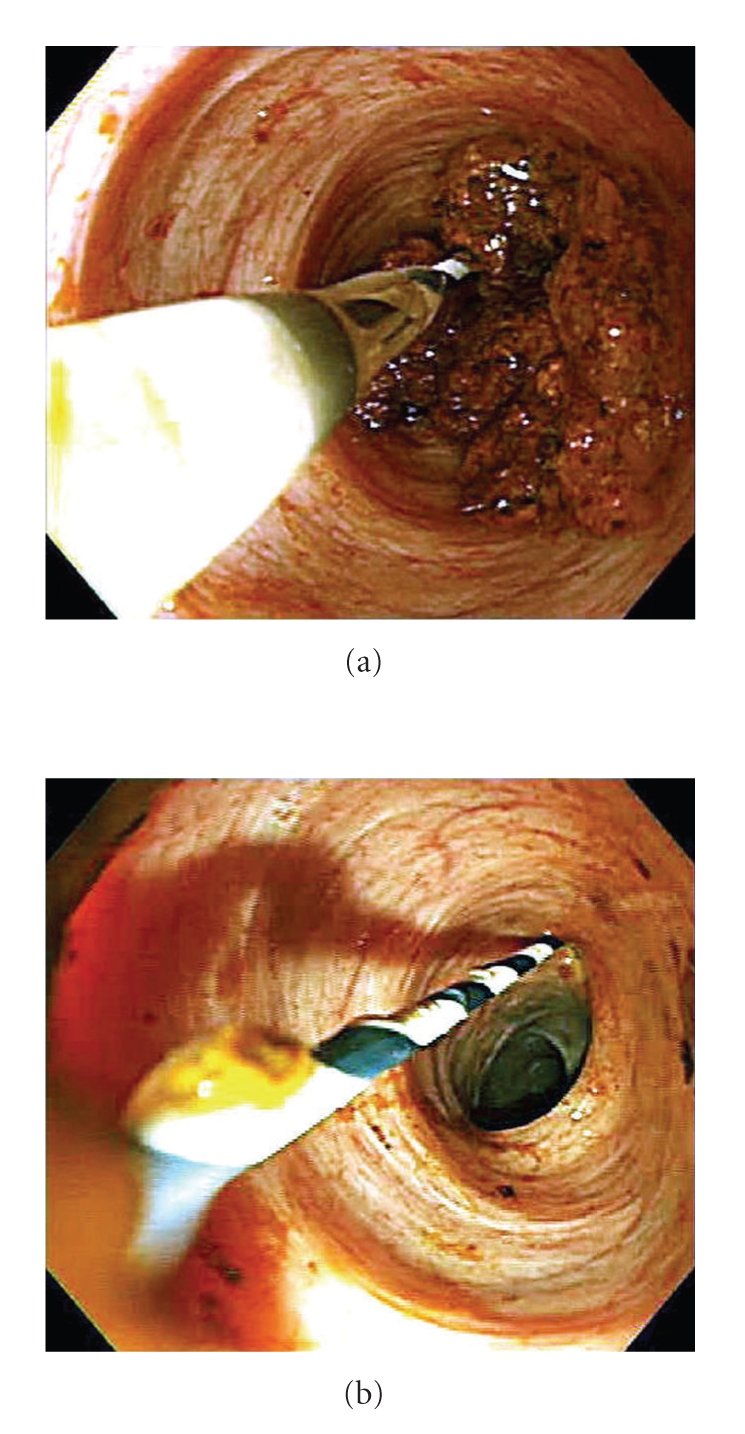
Direct cholangioscopy for bile duct stones. (a) Endoscopic imaging showed bile duct stones. (b) Bile duct stones were completely removed.

After papillary dilation using a 15-mm large-balloon (CRE Esophageal/Pyloric, length 5 cm, Boston Scientific Japan, Tokyo, Japan) without sphincterotomy because the major papilla was not well positioned for the sphincterotomy, an enteroscope as a direct cholangioscope was advanced into the bile duct. Direct endoscopic imaging revealed bile duct stones (Figure 3(a)). Bile duct stones were removed using a basket catheter and retrieval balloon. Finally, complete removal of bile duct stones was confirmed by direct endoscopic imaging (Figure 3(b)). Then, a guidewire was pulled out through the working channel. There was no procedure-related complication.

## 3. Discussion

ERCP has evolved into an essential therapeutic modality in patients with pancreaticobiliary diseases. Although successful cannulation of the bile duct is achieved in more than 90% of patients with normal gastrointestinal and biliary anatomy, ERCP in patients with surgically altered anatomy is challenging. Traditional ERCP in patients with a long-limb Roux-en-Y anastomosis is usually not feasible because of the inability to reach the papilla or biliopancreatic anastomosis with a standard side-viewing duodenoscope. A few skilled endoscopists have performed ERCP in such cases using pediatric or adult colonoscopes, or push enteroscopes [2], or by a percutaneous approach. However, despite using such special endoscopes and techniques, it was often impossible to reach the papilla or the biliopancreatic anastomotic site in patients with Roux-en-Y anastomosis. Furthermore, The acute angle of R-Y limb can be one of major factors of failed enteroscopy-assisted ERCP. When we encounter such patients, we usually change endoscopic therapy to alternative therapies, namely, lithotripsy via PTBD route or surgery. However, they are time-consuming or have relatively high risk of morbidity for elderly people. Recently developed balloon enteroscope systems have made it possible to reach the papilla or biliopancreatoenteric anastomosis site with certainty even in patients with Roux-en-Y surgical anastomoses. Furthermore, the present rendezvous technique for acute angle of R-Y limb could help easily scope insertion into the papilla. To our knowledge, this is the first report on the usefulness of rendezvous technique using single balloon enteroscope in such patients.

In the present case, we performed a large papillary balloon dilation technique without sphincterotomy because the major papilla was not wellpositioned for the sphincterotomy. The procedure of large balloon dilation performed after sphincterotomy has relatively been established for the removal of large bile duct stones without any serious complication [11–16]. Recently, latest article revealed that endoscopic papillary dilation using a large balloon was safe and effective in patients with normal anatomy and large bile duct stones though it was a retrospective analysis [17]. However, the outcome should be evaluated in the near future. Until then, sufficient care should be taken if we use this procedure. 

The direct peroral cholangioscopy using an ultraslim has been reported [18, 19]. In the present study, we could completely remove bile duct stones under directly endoscopic imaging using a standard balloon enteroscopy after papillary large-balloon dilation. Although the usefulness of a direct peroral cholagioscopy for the lithotripsy is controversial without performing electric hydraulic or laser lithotripsy, it may have some potential for confirming the residual stones because reintervention for residual stones is tough in patients with R-Y. 

In conclusion, although care has to be taken that procedure-related complications can occur and further study is needed, single balloon enteroscopy-assisted ERCP using a rendezvous technique may have a potential for selected patients.

## Abbreviations

ERCP: Endoscopic retrograde cholangiopancreatography
R-Y: Roux-en-Y anastomosis
SBE: Single-balloon enteroscopy
PTBD: Percutaneous transhepatic biliary drainage.
